# Optimizing adipose-derived stromal vascular fraction storage: Temperature and time impact on cell viability in regenerative medicine

**DOI:** 10.1097/MD.0000000000039859

**Published:** 2024-09-20

**Authors:** Darko Jović, Ljubiša Preradović, Filip Jović, Mićo Kremenović, Darko Lukić, Milica Antonić, Nikola Unčanin, Matija Jović

**Affiliations:** a University of Banja Luka, Faculty of Medicine, Banja Luka, Bosnia and Herzegovina; b Special Hospital S-tetik, Banja Luka, Bosnia and Herzegovina; c University of Ulm, Faculty of Medicine, Ulm, Germany; d University Clinical Centre of the Republic of Srpska, Banja Luka, Bosnia and Herzegovina; e University of Belgrade, Faculty of Medicine, Belgrade, Serbia.

**Keywords:** cell count, cell storage, cell survival, liposuction, stem cell therapy, stem cells, storage temperature, storage time, stromal vascular fraction

## Abstract

**Background:**

The adipose-derived stromal vascular fraction (SVF) plays a crucial role in regenerative medicine owing to its regenerative and immunomodulatory properties. However, the effective utilization of SVF in therapeutic applications requires careful consideration of storage conditions to maintain cell viability.

**Methods:**

We conducted a research on 43 patients of different ages and sexes who were older than 18 years. This study explored the impact of different temperatures (‐80, ‐20, and 4 °C) on SVF storage in platelet-poor plasma for 1 and 6 months. SVF extracted using a semi-UNISTATION™ system was subjected to rigorous analysis of cell count and viability using a LUNA-STEM™ Dual Fluorescence Cell Counter.

**Results:**

The results indicated a significant correlation between the storage conditions and SVF viability. Notably, storing SVF at 4 °C demonstrated the highest cell viability and count, while ‐80 °C storage exhibited the least favorable outcomes. This study emphasizes the importance of minimizing storage time to preserve SVF viability, as evidenced by a decline in both cell count and viability over a 6-month period. Comparisons with the existing literature underscore the need for precise protocols for SVF storage, with considerations for temperature and cryoprotective agents. These findings provide valuable insights for developing optimal SVF storage protocols to enhance therapeutic outcomes and reduce the need for repeated adipose tissue harvesting. Despite the limitations of the study, such as the use of a cell counter instead of flow cytometry, the results establish the foundation for further research on refining SVF storage methods.

**Conclusion:**

The ideal storage temperature is from 4 °C, while the length of storage time inversely affects the viability of SVF; the longer the storage time, the lower the number and the viability of SVF cells, regardless of the temperature at which they are preserved.

## 1. Introduction

The adipose-derived stromal vascular fraction (SVF) is important in cell treatment and tissue engineering because of its regenerative and immunomodulatory characteristics.^[1]^ SVF applications often involve a time delay or multiple applications at different time intervals, thus requiring SVF storage under certain temperature conditions and media that affect cell number and viability. In addition to temperature, the survival of SVF cells is significantly affected by storage time.

An ideal medium to store SVF should non to cells; that is, a non and chemically inert medium that provides a high survival rate and can be applied without previous washing.^[2,3]^ Some authors have used cryopreservation of SVF to increase cell survival. The most commonly used cryoprotectant is dimethyl sulfoxide (DMSO), which is cytotoxic.^[4,5]^ But the application of SVF in combination with autologous fat grafts indicates that the cryoprotectant dose can be significantly decreased, and the fat survival rate can be increased.^[6]^

In addition to stem cells, SVF also contains other cells such as endothelial precursor cells, endothelial cells, and macrophages, as well as numerous growth factors and cytokines, which, in addition to stem cells, decrease the inflammatory response, increase the regenerative capacity, and promote the healing of an injury and wound. Recent reports on the clinical application of SVF have ranked it equal to the application of stem cells, and even higher.^[7–15]^

The quality of the SVF substrate is very important, and is reflected in the ratio of the number of living and dead cells, which is defined as the viability, as well as the number of SVF cells; the higher the number, the higher the quality of the sample.

To date, we have focused on creating protocols regarding the administration of SVF, that is, stem cells, the concentration of SVF cells administered, and the SVF administration method used, which is the required number of repetitions of treatment. Often, SVF application in regenerative medicine, especially in the regeneration of articular surfaces, tendons, and connective sheaths, requires repetition at certain time intervals, thus requiring the best storage method for the obtained SVF to give a cell count and viability close to that obtained after fat harvesting and processing techniques.

The development of SVF storage protocol, that is, the optimum storage time and temperature certainly has a significant effect on the fat harvesting process, that is, liposuction, because in that case fat is harvested only once, and not several times as when administering freshly obtained SVF, and in that way it significantly affects the cost of the procedure itself, the morbidity of the donor site for fat grafting, as well as the discomfort reduction in patients who are exposed to long-term medical procedures.^[16]^

Many techniques are currently used worldwide to harvest adipose-derived SVF, ranging from the classic excision of adipose tissue using the Coleman technique to classic liposuction.^[17,18]^

Platelet-poor plasma (PPP) can be used as an alternative to DMSO because it contains growth factors, cytokines, and proteins that can support cell survival and proliferation. Therefore, we used this medium in our study to preserve SVF.

Unlike DMSO, which can have cytotoxic effects at higher concentrations, PPP is a biological fluid that is generally well-tolerated by cells. This makes it a potentially safe alternative for cell culture and preservation. DMSO remains a widely used cryoprotectant because it effectively prevents ice crystal formation during cell preservation.^[19]^

The objective of this study was to improve the development of a more precise protocol for the best storage of SVF, to apply it multiple times with the same quality, and to reduce expenses and inconvenience resulting from the multiple extractions of adipose tissue needed to obtain SVF.

## 2. Materials and methods

We started the research in February 2022 at the Special Hospital S-tetik and finished it in June 2023 on 43 patients of different ages and sexes. The research project included all patients older than 18 years who were familiar with the examination method and did not suffer from any serious diseases (malignant diseases, diabetes, heart diseases, etc). All the patients included in the study provided written informed consent.

All methods were performed in accordance with the relevant guidelines and regulations.

After injecting the tumescent solution, we obtained fat by classical vibration-assisted liposuction with 3 mm cannulae, which were placed through small incisions from different parts of the body (stomach, lumbar region, and upper legs).^[20,21]^

We used a tumescent solution containing 500 mg lidocaine, 1 mg epinephrine, and 12.5 mEq sodium bicarbonate per one liter of 0.9% saline solution. Special care was taken to ensure that the maximum dose of lidocaine did not exceed 55 mg/kg of the total amount of the tumescent substance. During liposuction, we used a negative pressure that was slightly lower than that typically used for liposuction and was between 300 and 400 mm Hg. This lower pressure helps reduce trauma to fat cells, which is crucial for preserving SVF integrity.

Vibration-assisted liposuction presents several advantages over conventional liposuction for obtaining the SVF, including enhanced efficiency, superior tissue preservation, and improved patient recovery. However, this technique also has notable disadvantages, such as increased costs, requirement for more advanced surgical training, and potential for fat tissue damage if the equipment is not utilized correctly, which may result in variability in the quality of the SVF obtained.

**Figure 1.**
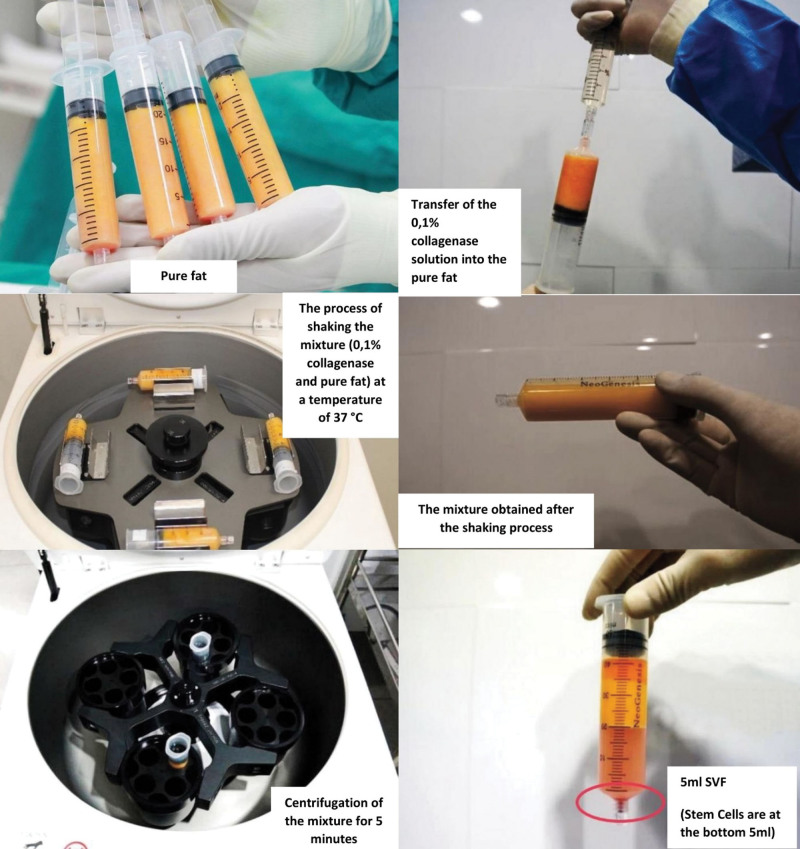
Harvesting adipose tissue and a process to obtain SVF from the adipose tissue. SVF = stromal vascular fraction.

The obtained fat was used to obtain SVF using the semiautomatic UNISTATION 2nd version system (NeoGenesis, Seoul, South Korea), which provides a considerable amount of sterile SVF with high reproducibility.^[22]^ Unistation is composed of a centrifuge, incubator, and shaker for the isolation, extraction, and cultivation of various cells. It is FDA-approved. In this process, 20 mL of centrifuged adipose tissue was combined with 20 mL of 0.1% collagenase and mixed at 37 °C for 30 minutes. The mixture was then centrifuged at 800 rpm for 5 minutes. Following centrifugation, 5 mL of SVF concentrate was carefully extracted from the lower part of the syringe. Subsequently, the concentrate was mixed with 5 mL PPP and 30 mL of 0.9% NaCl. The mixture underwent another round of centrifugation at 800 rpm for 5 minutes to neutralize collagenase and wash the SVF concentrate (Fig. 1).

The obtained SVF concentrate was stored in a NaCl 0.9% solution and PPP, where it was isolated at different temperatures.^[23]^ We cooled the resulting SVF suspension gradually at a rate of 1 °C per minute until we reached the desired temperature of 4, ‐20, and –80 °C. This is because slow cooling of the cells ensures less formation of ice crystals inside the cells, which increases the viability of SVF cells.^[24]^ After storage for 1 month, as well as for 6 months, we started the thawing process by placing the cryovial with frozen SVF from the freezer and placing it in a 37 °C water bath. After a few minutes, the SVF cells were thawed. In this way, we prevented prolonged thawing time, which could reduce the viability of SVF cells.^[25–27]^

**Figure 2.**
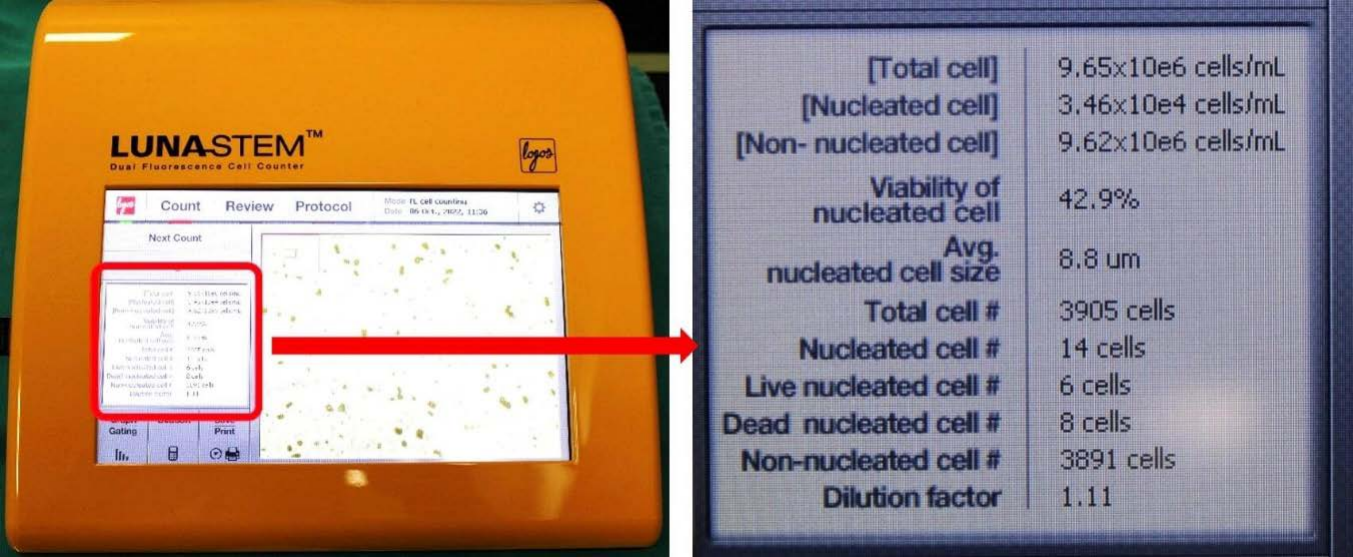
We determined the number and viability of SVF cells using LUNA-STEM™ Dual Fluorescence Cell Counter. SVF = stromal vascular fraction.

In this study, we determined the number and viability of SVF cells, which are nucleated cells, with the LUNA-STEM™ dual-fluorescence cell counter, which is an advanced device for analyzing the number and viability of SVF cells (Fig. 2). It uses acridine orange and propidium iodide to distinguish between live and dead nucleated cells, which represent SVF cells, and cells that are not stained represent non cells, such as red blood cells, embryonic stem cells, and microvascular elements.

**Figure 3.**
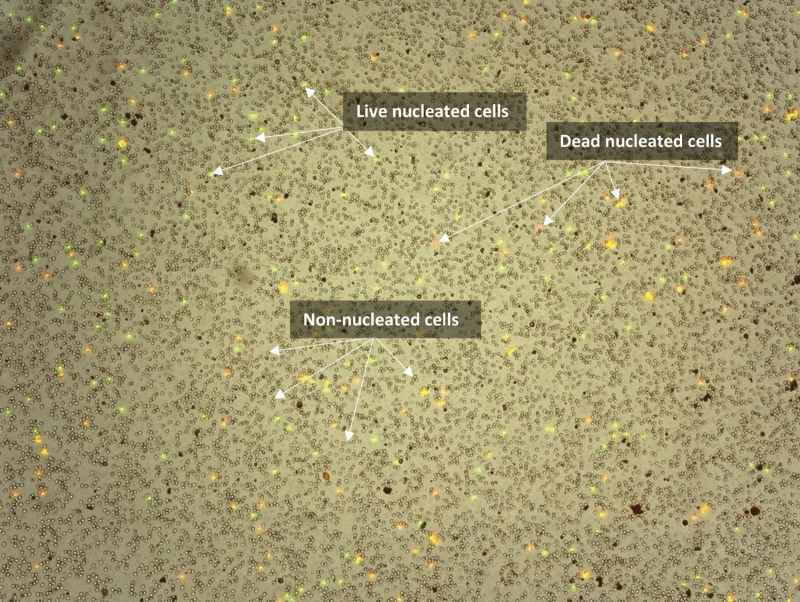
Dead nucleated cells are colored red while living nucleated cells are colored green.

This device enables high quality and sensitivity to stem cells and other SVF cells. Dead nucleated cells were colored red, whereas living nucleated cells were colored green (Fig. 3).

Cell count and viability were measured immediately after obtaining SVF and after maintaining them at different temperature conditions for certain periods to acquire data on the optimum SVF storage time and temperature.

The data were processed and analyzed using the Statistical Package for the Social Sciences version 23 using nonparametric tests (Friedman test and Wilcoxon signed ranks test) and Spearman correlation coefficient.

The statistical significance was *P* < .05.

## 3. Results

During the research, 43 SVF samples were analyzed for storage at different temperatures in different periods. The SVF sample obtained from each patient was divided into 3 groups of 2 sample containers that were stored at different temperatures (4, ‐20, and ‐80 °C) and taken for measurement at different time periods.

**Figure 4.**
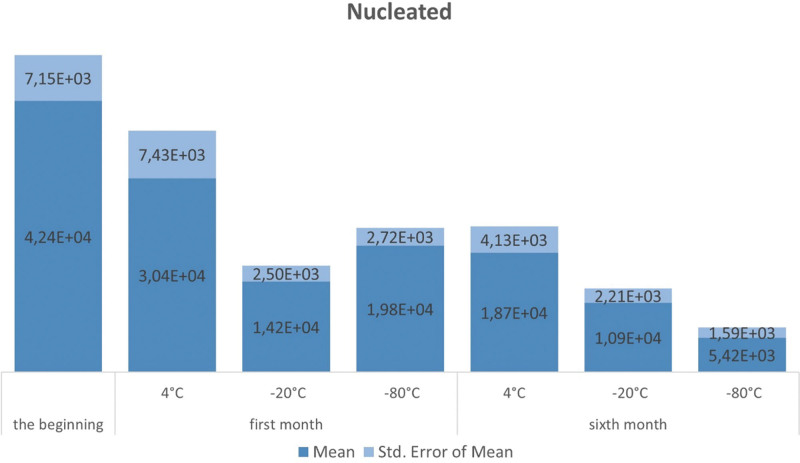
The number of nucleated cells on the day of obtaining SVF, 1 month, and 6 months after obtaining SVF at certain storage temperatures. SVF = stromal vascular fraction.

**Figure 5.**
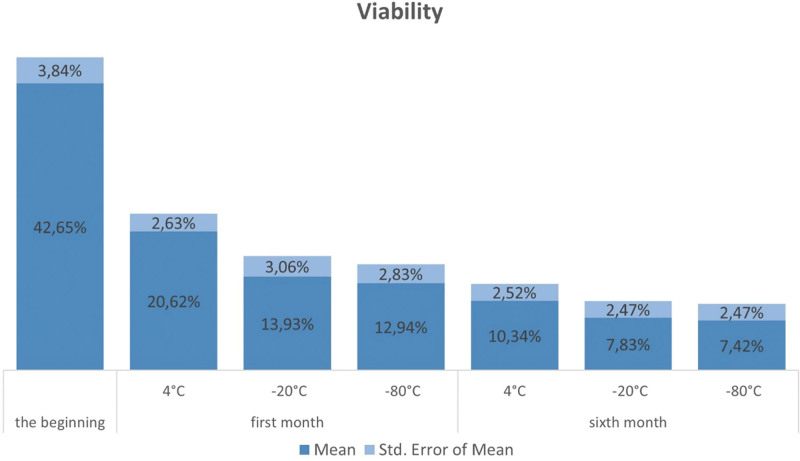
The viability of cells on the day of obtaining SVF, 1 month and 6 months after obtaining SVF at certain storage temperatures. SVF = stromal vascular fraction.

SVF samples were stored at 4, ‐20, and ‐80 °C for 6 months, and were directly frozen at temperatures of ‐20 and ‐80 °C without using any additional materials for cryopreservation. The SVF-nucleated cells and their viability were measured at the time of obtaining SVF, after 1 month, and after 6 months of storage at different temperatures (Figs. 4 and 5), respectively.

Sex structure: 37 (86%) female and 6 (14%) male.

Respondents’ ages ranged from 32 to 74 years, and the average age of the respondents was 50, 65 years.

Body fat: The body fat (BMI) of the respondents ranged from 23.05 to 43.38 kg/m^2^, with an average of 27.74 kg/m^2^.

Pearson correlation coefficient was used to compare the correlation of initial values with values after 1 and 6 months, and by testing the values of individual measurements at the same temperatures after 1 and 6 months (Tables 1 and 2), respectively.

**Table 1:** (Highly) significant values of Pearson correlation coefficient and (highly) statistically significant difference using nonparametric tests (Friedman test and Wilcoxon signed ranks test) for nucleated cells at different temperatures during 6 months.

Age	BMI	The beginning	The 1st month			The 6th month			*r*	*P* §	*χ* ^2^	*P* †	*z*	*P* ‡
			4 °C	‐20 °C	‐80 °C	4 °C	‐20 °C	‐80 °C						
✓	✓								0.606	.000				
✓							✓		‐0.509	.000				
	✓	✓							0.387	.010				
		✓				✓			0.494	.001				
			✓				✓		0.337	.027				
		✓	✓	✓	✓						13,727	.003		
		✓				✓	✓	✓			13,727	.003		
		✓	✓			✓					13,568	.001		
		✓		✓			✓				24,874	.000		
		✓			✓			✓			45,527	.000		
						✓	✓	✓			10,533	.005		
		✓		✓									‐3847	.000
		✓				✓							‐3777	.000
		✓					✓						‐4293	.000
		✓						✓					‐5196	.000
					✓			✓					‐4102	.000
						✓		✓					‐2922	.003
		✓	✓										‐2307	.021
		✓			✓								‐2326	.020
			✓	✓									‐2339	.019
							✓	✓					‐2284	.022

BMI = body mass index.

§ Pearson correlation.

† Friedman test.

‡ Wilcoxon signed ranks test.

**Table 2:** (Highly) significant values of Pearson correlation coefficient and (highly) statistically significant difference using nonparametric tests (Friedman test and Wilcoxon signed ranks test) for viability at different temperatures during 6 months.

Age	BMI	The beginning	The first month			The sixth month			*r*	*P* §	*χ* ^2^	*P* †	*z*	*P* ‡
			4 °C	‐20 °C	‐80 °C	4 °C	‐20 °C	‐80 °C						
✓		✓							‐0.411	.006				
✓							✓		‐0.411	.006				
		✓	✓						0.513	.000				
✓					✓				‐0.362	.017				
✓								✓	‐0.351	.021				
	✓				✓				‐0.313	.041				
		✓		✓					0.319	.037				
				✓			✓		0.325	.033				
		✓	✓	✓	✓						45,660	.000		
		✓				✓	✓	✓			60,169	.000		
		✓	✓			✓					40,585	.001		
		✓		✓			✓				42,545	.000		
		✓			✓			✓			36,893	.000		
			✓	✓	✓						6088	.048		
		✓	✓										‐4824	.000
		✓		✓									‐5028	.000
		✓			✓								‐4394	.000
		✓				✓							‐4982	.000
		✓					✓						‐5340	.000
		✓						✓					‐5003	.000
			✓			✓							‐2577	.009
			✓		✓								‐2007	.045

BMI = body mass index.

§ Pearson correlation.

† Friedman test.

‡ Wilcoxon signed ranks test.

*The checkmarks in the tables indicate the parameters that were tested or compared using the appropriate statistical methods. For example, for the parameters age and BMI, a checkmark signifies that the correlation between a subject’s age and BMI was tested, with the resulting correlation coefficient presented in the “r” column, and statistically significant correlations denoted in the “P” column as “P§.”*

Table 1 *presents the number of nucleated cells initially measured (immediately following extraction from adipose tissue) and the corresponding cell counts at 1 and 6 months under different storage temperatures: 4, ‐20, and ‐80 °C. The Friedman test was employed for statistical analysis, with the resulting values displayed in the “χ²” column, and statistically significant differences indicated in the “P†” column.*

*If a statistically significant difference is identified using the Friedman test, pairwise comparisons between 2 parameters (from the 3 or 4 previously tested with the Friedman test) are conducted using the Wilcoxon signed-rank test. The resulting values are presented in the “z” column, with statistical significance denoted in the “P‡” column.*

## 4. Discussion

In many therapeutic procedures in which SVF is applied, it is necessary to repeat the procedure at different periods to achieve the best possible result. SVF is known that SVF has a variety of applications in clinical medicine, and numerous studies have made its application even more accessible and practical.^[28–31]^ SVF can create numerous growth factors and cytokines that affect the speed of chronic wound healing, reduce inflammatory reactions, and promote the formation of granulation tissue and vascularization, increasing its therapeutic importance.^[32]^ Rigotti et al repeatedly applied adipose-derived SVF to chronic wound areas and observed ultrastructural improvements in the quality of the tissue itself as well as new vascular formation and clinical improvement.^[33,34]^

Repeating adipose tissue harvesting procedures to obtain SVF can cause adverse effects, including higher costs, unfavorable appearance, increased patient morbidity, and discomfort, and can be a limiting factor for immediate use.^[3,35,36]^

By storing SVF under certain subcooling conditions, we can avoid the morbidity associated with repeated liposuction, allowing multiple uses of well-preserved SVF solution after its primary collection. Currently, the standard SVF subcooling temperature is not yet fully known, and many studies are addressing this problem to create the best storage protocol and administration of SVF.^[7]^

Our study showed that the number and viability of SVF cells were highest immediately after collection. We found that the least decline in both number and viability occurred when the cells were stored at 4 °C, whereas the most significant decrease was observed at ‐80 °C. Statistically significant differences were noted in the preservation of SVF cells when comparing storage at 4, ‐20, and ‐80 °C after 1 month, and these differences remained evident after 6 months.

Although our work showed that a temperature of 4 °C is the best for storing SVF cells, it is important to emphasize that the viability of SVF cells drops by 50% after 1 month of storage at the aforementioned temperature, that is, from 42.65% to 20.62%.

Our data showed that the storage time of SVF greatly affected the number and viability of cells; thus, both the number and viability were higher at all measured temperatures 1 month after obtaining SVF related to a 6-month period, which implied that storage time affected the reduction in SVF number and viability.

Our research is similar to that of a group of authors who stated that the optimal storage temperature for SVF depends on the storage duration and specific temperature conditions. For short-term storage, usually up to a few days, maintaining SVF at 4 °C (refrigerator temperature) is ideal for preserving cell viability and function with minimal loss. For long-term storage, from months to a year, SVF is best stored at ‐80 °C or, more commonly, in liquid nitrogen at ‐196 °C.^[37–39]^

Dewi et al obtained interesting results when examining the viability and number of SVF cells; they divided the SVF obtained from adipose tissue into 2 groups. One SVF group was kept at room temperature, while the other group was stored at a temperature from 4 to 8 °C, and the number of SVF cells and their viability in were period, 4, 5, 6, 7, and 12 days. Thus, they concluded that the viability stored at a temperature from 4 to 8 °C remained higher than 75% but dropped 40% at room temperature; however, the number of SVF cells was higher at room temperature compared to the samples stored at 4 to 8 °C.^[40]^

In previous research, the data on how the viability of SVF after being obtained from adipose tissue and stored at room temperature decreased by 11.6%.^[35]^ Meanwhile, when stored frozen in autologous serum using cryopreservation with DMSO, not only the minimal decline in SVF cell viability but also their clonogenic ability and differentiation potential occur within 2 months.

No significant drop in viability was noted after storing the frozen SVF even after a year.^[41]^

Svalgaard et al obtained interesting results when storing SVF cells at temperatures: 4 °C, room temperature (18–20 °C), and 37 °C, while monitoring the viability of cells and proliferation potential of adipose-derived stromal cells. They concluded that the storage period and temperature of SVF cells negatively affected the number and viability of SVF cells; that is, the longer the storage time and the higher the storage temperature, the lower the number and viability of the cells.^[1]^

Pratiwi et al investigated the impact of temperature and storage media on umbilical cord-derived mesenchymal stem cells and SVF. At room temperature, both storage media exhibited a significant decrease in cell sustainability compared with cold storage (2–8 °C), where sustainability remained more stable. The highest sustainability for umbilical cord-derived mesenchymal stem cells at room and cold temperatures was achieved in the 0.9% NaCl solution (77% and 93%, respectively). SVF showed superior stability in autologous serum at cold temperatures (90.4%) compared to room temperature, with resilience declining from 96% on day 1 to 77% on day 6.^[22]^

This study has several limitations. The first one refers to the use of the LUNA-STEM Dual Fluorescence Cell Counter that delivers cell count and determines the viability of SVF cells, unlike flow cytometry that provides a detailed analysis of all the cells constituting the SVF, that is, determines the number and the characteristics of stem cells, endothelial precursor cells, endothelial cells, macrophages and other SVF cells.

Another limitation of this study is that we did not examine the impact of different environments on storing SVF cells or the application of materials for cryopreservation, enumeration, and SVF cell viability.

## 5. Conclusions

The conclusion is clear based on our results: both the length of the storage time and the temperature at which SVF samples are stored influence the number and viability of cells. The ideal storage temperature is from 4 °C, while the length of storage time inversely affects the viability of SVF; the longer the storage time, the lower the number and the viability of SVF cells, regardless of the temperature at which they are preserved. The least favorable temperature for SVF storage was ‐80 °C, at which the viability and the number of cells were decreased to the highest degree compared to temperatures from ‐20 and 4 °C.

## Author contributions

**Conceptualization:** Darko Jović, Filip Jović.

**Data curation:** Ljubiša Preradović, Milica Antonić.

**Formal analysis:** Darko Jović, Filip Jović, Nikola Unčanin, Matija Jović.

**Funding acquisition:** Darko Jović.

**Investigation:** Darko Jović, Filip Jović, Mićo Kremenović, Darko Lukić.

**Methodology:** Darko Jović, Ljubiša Preradović.

**Project administration:** Darko Jović.

**Resources:** Darko Jović, Milica Antonić, Matija Jović.

**Software:** Ljubiša Preradović, Nikola Unčanin, Matija Jović.

**Supervision:** Darko Jović, Filip Jović.

**Validation:** Darko Jović, Darko Lukić.

**Visualization:** Darko Jović, Filip Jović.

**Writing – original draft:** Darko Jović.

**Writing – review & editing:** Ljubiša Preradović, Filip Jović, Mićo Kremenović, Matija Jović.
